# Deconvolution of cellular subsets in human tissue based on targeted DNA methylation analysis at individual CpG sites

**DOI:** 10.1186/s12915-020-00910-4

**Published:** 2020-11-24

**Authors:** Marco Schmidt, Tiago Maié, Edgar Dahl, Ivan G. Costa, Wolfgang Wagner

**Affiliations:** 1 Helmholtz-Institute for Biomedical Engineering, Stem Cell Biology and Cellular Engineering, RWTH Aachen University Medical School, 52074 Aachen, Germany; 2 Institute for Biomedical Engineering – Cell Biology, University Hospital of RWTH Aachen, 52074 Aachen, Germany; 3 Institute for Computational Genomics, Joint Research Center for Computational Biomedicine, RWTH Aachen University Medical School, 52074 Aachen, Germany; 4 RWTH centralized Biomaterial Bank (RWTH cBMB), Medical Faculty, RWTH Aachen University, Aachen, Germany

**Keywords:** Cell types, Deconvolution, DNA methylation, Epigenetic, Human, Fibrosis, Cancer, CpG, Pyrosequencing, NNLS

## Abstract

**Background:**

The complex composition of different cell types within a tissue can be estimated by deconvolution of bulk gene expression profiles or with various single-cell sequencing approaches. Alternatively, DNA methylation (DNAm) profiles have been used to establish an atlas for multiple human tissues and cell types. DNAm is particularly suitable for deconvolution of cell types because each CG dinucleotide (CpG site) has only two states per DNA strand—methylated or non-methylated—and these epigenetic modifications are very consistent during cellular differentiation. So far, deconvolution of DNAm profiles implies complex signatures of many CpGs that are often measured by genome-wide analysis with Illumina BeadChip microarrays. In this study, we investigated if the characterization of cell types in tissue is also feasible with individual cell type-specific CpG sites, which can be addressed by targeted analysis, such as pyrosequencing.

**Results:**

We compiled and curated 579 Illumina 450k BeadChip DNAm profiles of 14 different non-malignant human cell types. A training and validation strategy was applied to identify and test for cell type-specific CpGs. We initially focused on estimating the relative amount of fibroblasts using two CpGs that were either hypermethylated or hypomethylated in fibroblasts. The combination of these two DNAm levels into a “FibroScore” correlated with the state of fibrosis and was associated with overall survival in various types of cancer. Furthermore, we identified hypomethylated CpGs for leukocytes, endothelial cells, epithelial cells, hepatocytes, glia, neurons, fibroblasts, and induced pluripotent stem cells. The accuracy of this eight CpG signature was tested in additional BeadChip datasets of defined cell mixtures and the results were comparable to previously published signatures based on several thousand CpGs. Finally, we established and validated pyrosequencing assays for the relevant CpGs that can be utilized for classification and deconvolution of cell types.

**Conclusion:**

This proof of concept study demonstrates that DNAm analysis at individual CpGs reflects the cellular composition of cellular mixtures and different tissues. Targeted analysis of these genomic regions facilitates robust methods for application in basic research and clinical settings.

## Background

The human body comprises hundreds of different cell types, but a clear and commonly accepted classification is still elusive [1]. The cellular characterization is usually based on ontogenetic origin within a tissue, cellular morphology, and particularly on expression of cell type-specific surface markers. These markers can also be used to isolate and purify distinct cellular subsets, e.g., by flow cytometry upon labeling with specific antibodies. However, most cell types do not have a unique panel of surface markers and bulk analysis without physical sorting masks the contribution of rare cell types [2, 3]. In the advent of single-cell omics data, e.g., by transcriptomics, ATAC-seq, or even single-cell proteomics, it is possible to discern between cells by molecular means on a cell-by-cell basis [4]. However, these methods require fresh material, they are relatively expensive, and clear demarcation of cell types remains a challenge. Alternatively, it is possible to use transcriptomic or epigenetic bulk data to estimate the cellular composition in tissues based on deconvolution algorithms [2, 5–8]. Better insight into the composition of cell types may support pathological assessment, target identification, and staging of various diseases [6, 9]. To this end, a robust, simple, and cost-effective method to estimate the cellular composition in a given tissue sample would be advantageous.

DNA methylation (DNAm) at CG dinucleotides (CpGs) is a stable and heritable modification that is directly linked to cellular differentiation [9–11]. It can be analyzed quantitatively on single base resolution and—in contrast to gene expression—every cell has only two alleles, which makes DNAm ideally suited for deconvolution approaches [12]. Amongst the first applications was the estimation of leukocyte subsets in blood [5]. More recently, it has been shown that comprehensive human cell type DNAm profiles facilitate the estimation of the origin of circulating cell-free DNA [7]. Deconvolution may either be based on a reference dataset, or it can be trained reference-free [9, 13, 14]. So far, epigenetic deconvolution was mostly based on genome-wide DNAm profiles, generated by the Illumina BeadChip technology. This method is relatively cost-effective and provides a very broad insight into genome-wide DNAm patterns. However, targeted methods for DNAm analysis, such as pyrosequencing of specific CpGs, may facilitate faster and even more cost-efficient analysis with less starting material, while reducing batch to batch variation and other technical challenges [15]. We have recently developed targeted DNAm signatures for pyrosequencing of individual CpGs to achieve deconvolution of leukocyte subsets that correlate with conventional blood counts [16]. In this study, we followed the hypothesis that the relative proportion of fibroblasts or even the complex cellular composition of human tissues can be estimated by targeted analysis of DNAm at individual CpGs.

## Results

### Compilation of global DNAm profiles of different cell types

**Fig. 1:**
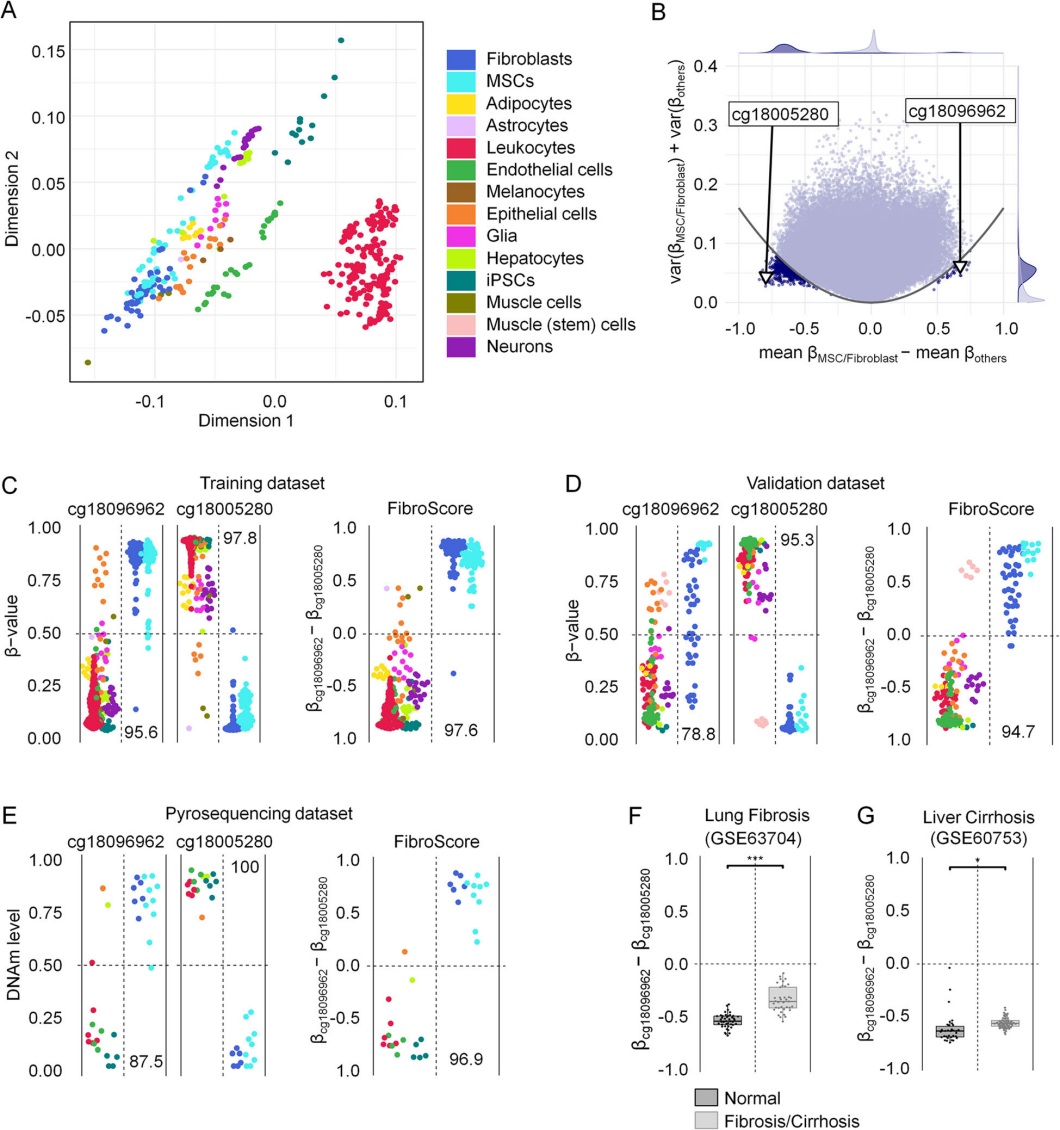
Selection of cell type-specific CpGs for fibroblasts. **a** Multidimensional scaling (MDS) plot of the training data set (*n* = 409) demonstrates that samples cluster by cell type across different studies. All CpGs shared between the 450K and EPIC BeadChip were considered (except XY chromosomes). **b** Differential mean DNAm levels of fibroblasts/MSCs versus all other cell types were plotted against the sum of variances within both groups. The CpGs, which have been selected for the FibroScore, are indicated. **c** DNAm levels (*β* values) of the two selected CpGs of the FibroScore in the training set. Numbers correspond to classification accuracy in percentage values. **d** DNAm levels of the two selected CpGs and the FibroScore for the validation set. Only muscle stem cells, which might closely resemble MSCs, were classified with fibroblasts/MSCs. Numbers correspond to classification accuracy in percentage values. **e** DNAm levels of the two selected CpGs and the FibroScore as determined by pyrosequencing in samples of different cell types. Almost all cell preparations (with exception of the HaCat cell line) were classified correctly. **f** The FibroScore is significantly higher in lung fibrosis versus healthy control tissue (GSE63704; 450K data) [62]. ***p < 0.001. **g** The FibroScore is significantly higher in liver cirrhosis versus healthy control tissue (GSE60753; 450K data) [29]. *p < 0.05

To identify cell type-specific CpGs for targeted methylation assays and tissue deconvolution, we curated and compiled 579 samples from 46 different studies, mostly generated with the Illumina 450K BeadChip technology. We only considered non-malignant samples and retrieved datasets of the following purified and characterized human cell types: fibroblasts, mesenchymal stromal cells (MSCs), adipocytes, astrocytes, leukocytes, endothelial cells, melanocytes, epithelial cells, glia, hepatocytes, muscle cells, muscle stem cells, neurons, and induced pluripotent stem cells (iPSCs). Four hundred nine samples were used as a training set and 170 samples from independent studies were used as a validation set (Additional file 1: Fig. S1A and Table S1) [7, 17–61]. Multidimensional scaling (MDS) of genome-wide DNAm profiles revealed that samples of the same cell type cluster together across different studies, supporting the notion that the cell type has major impact on DNAm patterns (Fig. 1a; Additional file 1: Fig. S1B).

### DNA methylation at fibroblast-associated CpGs can be indicative of fibrosis

Initially, we selected CpGs that might discern fibroblasts from other cell types. Such fibroblast-specific DNAm patterns could reflect the relative proportion of fibroblasts, for example for staging of fibrotic diseases. In our previous work, we addressed differences in DNAm profiles of fibroblasts versus MSCs, albeit classification of these cell types is hardly reflected by clear functional or molecular characteristics [63]. This is also reflected by their close relationship in the MDS plot. Therefore, we have decided to group both cell types together into the fibroblast category for subsequent analysis. To select fibroblast-specific CpGs that are either characteristically methylated or unmethylated in fibroblasts, we filtered for CpGs based on (1) the highest difference in mean DNAm in fibroblasts versus other cells and (2) small variance in DNAm levels within each of the two groups (Fig. 1b). CpG candidates were evaluated in terms of classification performance and ranked based on results from a 10-fold cross-validation setup. Based on this, we selected cg18096962 (associated with the lncRNA *RP11-60A8.1*) as hypermethylated and cg18005280 (associated with the gene leucine rich repeats and immunoglobulin like domains 1 [*LRIG1*]) as hypomethylated CpG site (Additional file 1: Fig. S1C,D). The difference in DNAm levels between these CpGs ([*β* value at cg18096962] − [*β* value at cg18005280]), referred to as FibroScore, could clearly distinguish fibroblasts from most other cell types (Fig. 1c, d). Only muscle stem cells, which have been differentiated for 24 h towards the myogenic lineage and might therefore closely resemble MSCs, were classified in the fibroblast category [50]. To further validate applicability of these CpG sites for targeted DNAm analysis, we analyzed DNA samples from cultured cells, frozen blood, and commonly used cell lines with pyrosequencing (Fig. 1e). Only one immortalized cell line was misclassified by the FibroScore: HaCat (spontaneously transformed keratinocytes for epithelial cells), which might be due to aberrant DNAm patterns by malignant transformation. Thus, targeted analysis of the two CpGs might be indicative of the fraction of fibroblasts/MSCs in tissue. In fact, when we applied the FibroScore to Illumina BeadChip datasets of lung fibrosis (GSE63704, Fig. 1f; Additional file 1: Fig. S1E) and liver cirrhosis (GSE60753, Fig. 1g; Additional file 1: Fig. S1F), we observed a significantly higher FibroScore in the fibrotic tissues as compared to healthy controls (two-sided *t* test: *p* = 2.51 × 10^−12^, and *p* = 0.0396, respectively) [29, 62].

### FibroScore correlates with overall survival in various types of cancer

**Fig. 2:**
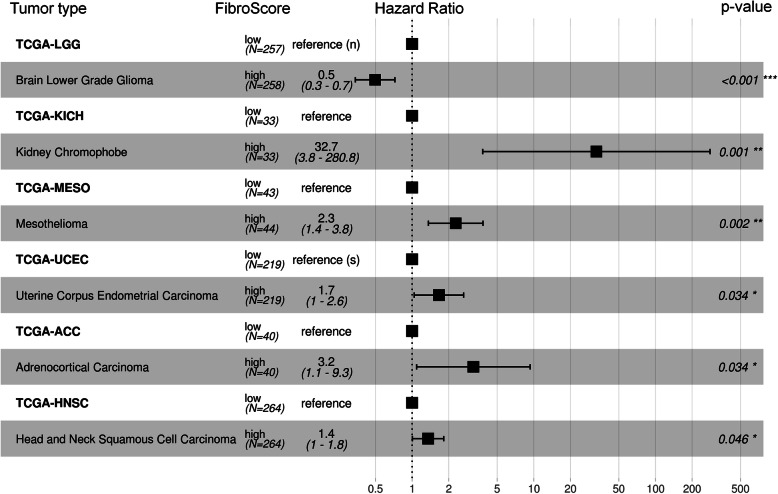
The FibroScore is associated with overall survival in several types of cancer. Hazards ratios from Cox proportional hazards models for datasets from The Cancer Genome Atlas (TCGA). Depicted are six types of cancer for which there is a significant difference in overall survival for patients with either high or low FibroScore. Unless specified otherwise, models take into account sex, age, tumor stage, and the FibroScore stratified by the median (450K BeadChip data). If some of these parameters were not available, we indicated missing cofactors next to the reference: s = sex, a = age, and n = stage

Cancer-associated fibroblasts (CAFs) determine the tumor microenvironment and play a crucial role for progression of malignancies [64]. Therefore, we anticipated that the FibroScore might also be of prognostic relevance for various types of cancers. To address this question, we utilized 32 datasets from The Cancer Genome Atlas (TCGA) and determined the FibroScore based on the DNAm at the two relevant CpGs. For each cancer type, the patient data was then stratified by the median FibroScore. A higher FibroScore was indicative of a significantly worse overall survival in chromophobe renal cell carcinoma (TCGA-KICH, *p* = 0.001), mesothelioma (TCGA-MESO, *p* = 0.002), uterine corpus endometrial carcinoma (TCGA-UCEC, *p* = 0.034), adrenocortical carcinoma (TCGA-ACC, *p* = 0.034), and head and neck squamous cell carcinoma (TCGA-HNSC, *p* = 0.046) (Fig. 2). Brain lower-grade glioma showed a significantly better survival outcome in patients with a higher FibroScore (TCGA-LGG, *p* = < 0.001). These results are reflected in the cox-proportional hazards adjusted survival curves for FibroScore (Additional file 1: Fig. S2). For all other cancer types, the stratification by the median FibroScore did not reveal a significant association with overall survival (Additional file 1: Fig. S3). These results support the notion that the DNAm at fibroblast-associated CpGs might be indicative of the fraction of CAFs, which is relevant for progression of various types of cancer.

### Deconvolution of cell types based on individual cell type-specific CpGs

**Fig. 3:**
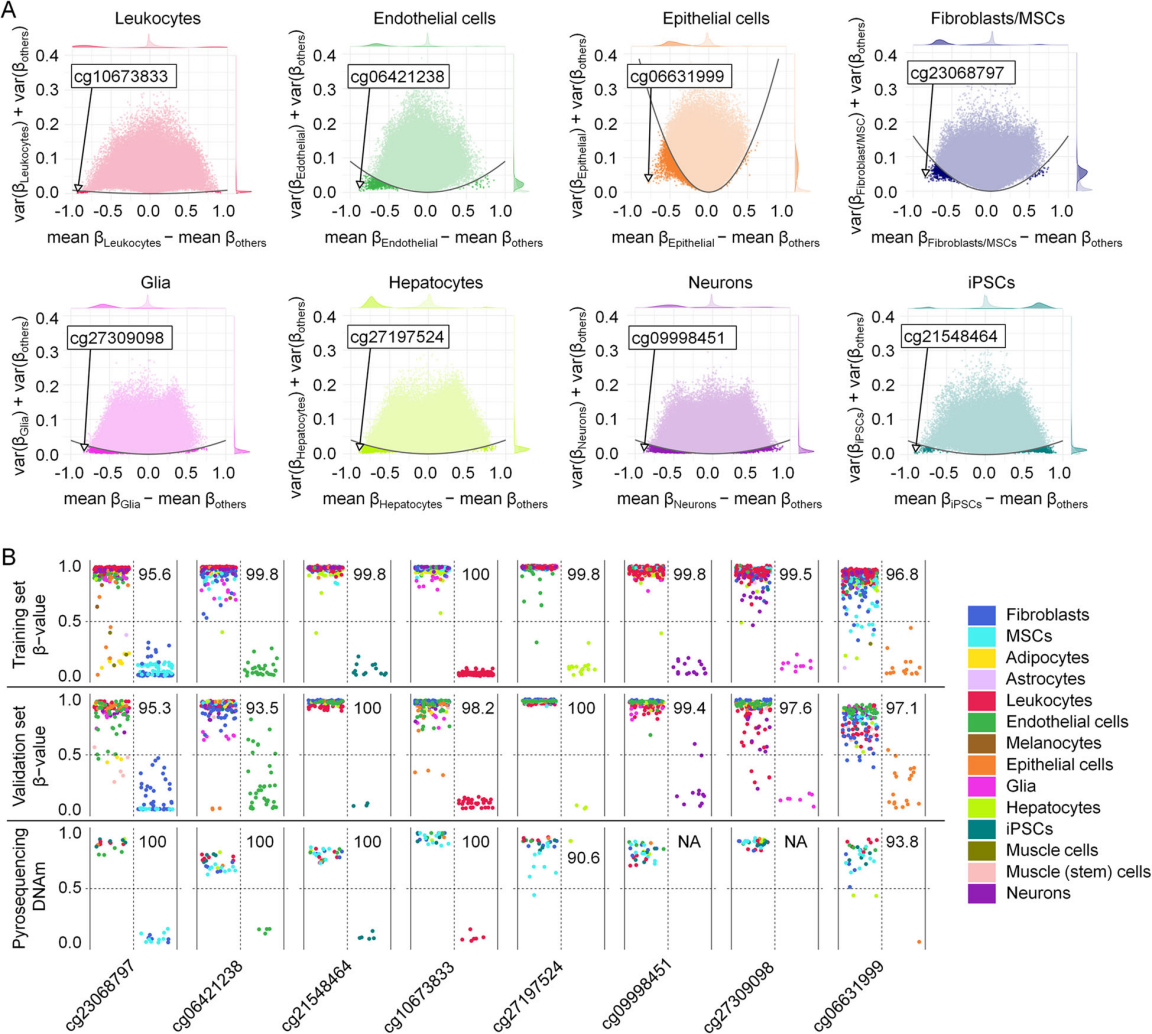
Cell type-specific CpG sites are preferentially hypomethylated. **a** Selection of cell type-specific CpGs for leukocytes, endothelial cells, epithelial cells, fibroblasts/MSCs, glia, hepatocytes, neurons, and iPSCs. The difference of mean *β* values of each cell type versus all other cell types was plotted against the sum of variances within both groups. CpGs for subsequent deconvolution are highlighted. **b** DNAm levels of the eight selected CpGs in the training, validation, and pyrosequencing datasets. The vast majority of samples revealed the expected cell type-specific hypomethylation, albeit pyrosequencing of liver cell lines (Hep3B and HuH-7) did not reveal hypomethylation at cg27197524 as expected for primary cells. Glia and neuron samples were not available for pyrosequencing. Numbers correspond to classification accuracy in percentage values

Subsequently, we followed the question if targeted analysis of individual CpGs might also reflect the composition of tissues. To this end, we have identified characteristic CpG sites for additional cell types using a similar procedure of CpG selection as mentioned above (difference in mean DNAm, variance in DNAm levels and classification performance). Notably, for all cell types—except for iPSCs, which resemble a ground state of non-differentiated cells—we identified more hypomethylated than hypermethylated CpGs in our feature selection (Fig. 3a). One hypomethylated CpG site was selected for every cell type, most of which were within introns and exons of corresponding genes (Additional file 1: Fig. S4): cg23068797 (*DNM2*, dynamin-2) for fibroblasts, cg10673833 (*MYO1G*, myosin IG) for leukocytes, cg06631999 (*STMN1*, stathmin) for epithelial cells, cg27197524 (*POLE*, DNA polymerase epsilon catalytic subunit A) for hepatocytes, cg06421238 (*WSCD1*, WSC domain-containing protein 1) for endothelial cells, cg27309098 (*AGAP1*, Arf-GAP with GTPase, ANK repeat and PH domain-containing protein 1) for glia, cg09998451 (*RAB3A*, ras-related protein Rab-3A) for neurons, and cg21548464 (lncRNA *DLEU1*, deleted in lymphocytic leukemia 1) for iPSCs [65, 66]. Cell type-specific hypomethylation was validated with the Illumina BeadChip data from the validation set and by pyrosequencing of various cell types and tissues (Fig. 3b). To estimate if the cell type-specific differential DNAm might also be reflected in gene expression levels, we utilized the Primary Cell Atlas [67]. In fact, *MYO1G, WSCD1, RAB3A*, and *DLEU1* seemed to reveal cell type-specific upregulation, albeit only the CpG for *MYO1G* was located in the promoter region (Additional file 1: Fig. S5).

**Fig. 4:**
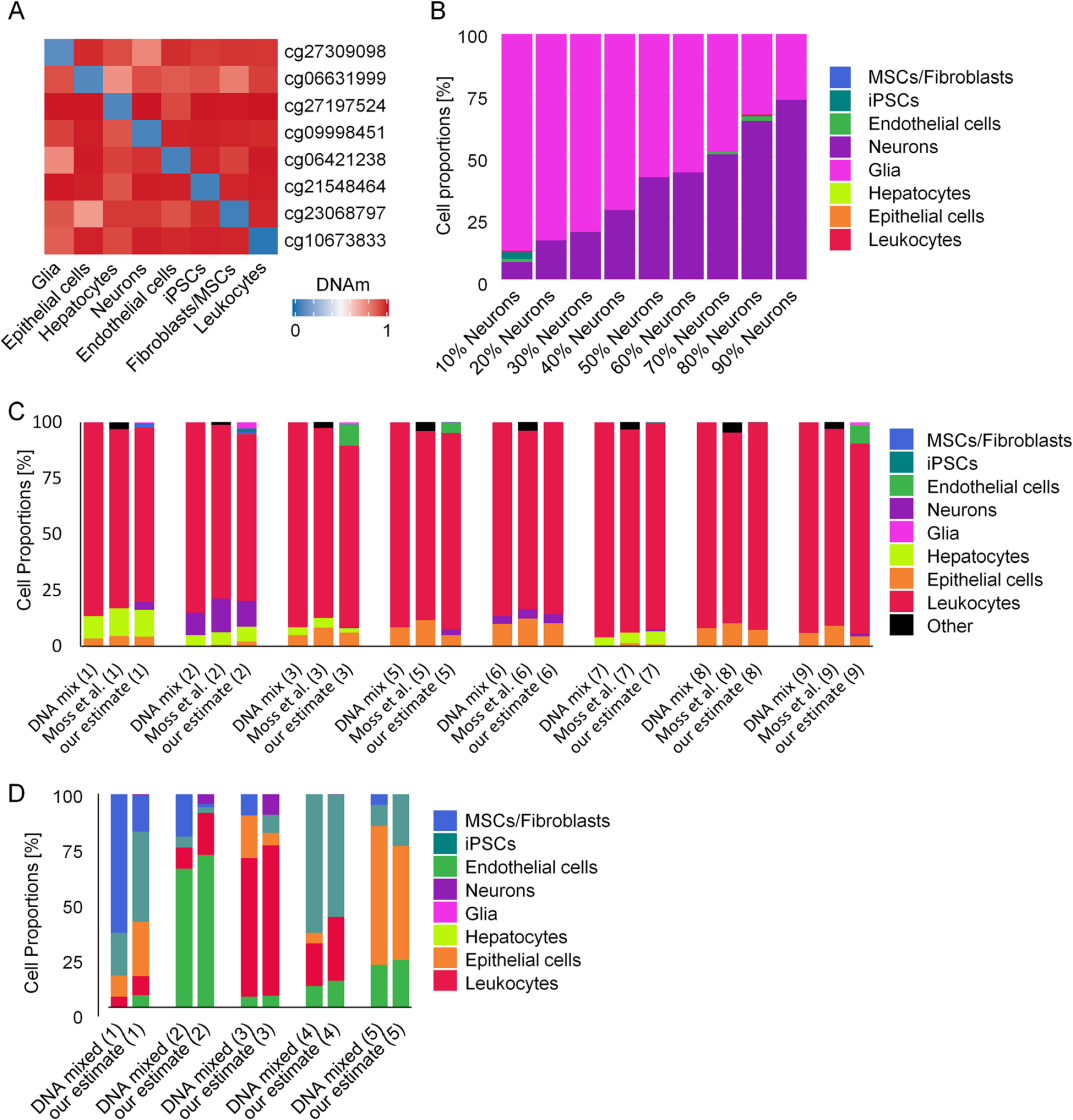
Deconvolution of cell mixtures based on individual cell type-specific CpGs. **a** Heatmap of mean *β* values of the reference matrix (450K data of the training set), which is used for deconvolution. **b** Deconvolution of in vitro neuron-glia-DNA-mixes from dataset GSE41826 [68]. The predicted cell fractions by our NNLS-based deconvolution with eight CpGs are depicted. **c** Deconvolution of eight different in vitro DNA mixes from dataset GSE122126 [7]. The real composition of DNA fractions is plotted next to the predictions by the signatures of Moss et al. (estimates for leukocyte subsets, epithelial cells, and others were combined). The estimates with our NNLS model closely resembled the DNA mixtures of different cell types. Data for DNA mix 4 was lacking one of the eight CpGs and was therefore excluded. **d** Deconvolution of in vitro DNA mixes measured with pyrosequencing. Five different mixes of five different cell types in different proportions were measured at the eight different sites. Shown are mixed versus estimated cellular fractions with our NNLS-based deconvolution

We used the mean DNAm levels for the selected CpGs in eight distinct cell types in the training dataset as our reference matrix when applying the non-negative least squares (NNLS) deconvolution algorithm (Fig. 4a; Additional file 2). The NNLS algorithm could then be used to generate estimates for the cellular composition of tissues and DNA mixes, based on the DNAm of eight CpGs. To assess the performance of our deconvolution model, we used a 450K Illumina BeadChip dataset of neuron-glia-DNA-mixes in incremental proportions [68]. Our predictions correlated very well with the neuronal/glial proportions, with only a small fraction of other cell types being predicted as present (Fig. 4b; Additional file 1: Fig. S6A). Alternatively, we tested non-negative matrix factorization (NMF) and EpiDISH, which performed not as well as the NNLS approach (Additional file 1: Fig. S6A,B) [69, 70]. Next, we tested the deconvolution performance on 450K data from in vitro DNA mixes of various different cell types [7]. Our training set did not include categories for lung and colon epithelial cells and therefore we assigned them to our epithelial cell category. Again, the predictions of our NNLS approach overall closely represented the real composition of cell types and the results were similar to the previously described deconvolution results that considered about 6000 CpGs [7] (Fig. 4c; Additional file 1: Fig. S6C). We then tested if deconvolution of different cell types would also be feasible by targeted methods. Therefore, we prepared five in vitro DNA mixes of five different cell types in varying proportions and analyzed all cell type-specific CpGs by pyrosequencing. The estimated composition closely resembled the previously mixed fractions (Fig.4d; Additional file 1: Fig. S6D).

Validation of our deconvolution approach for complex tissue was hampered by the availability of DNAm profiles for samples with a defined composition of cell types. Therefore, we applied our deconvolution to various non-malignant tissue samples from TCGA (Additional file 1: Fig. S7A). Overall, the estimates for the different cell types are compatible with the assumed real cellular composition of the tissue. Furthermore, we have also applied our targeted pyrosequencing approach to various DNA samples from tissues, and the predictions indicated similar composition of different cell types as estimated for the Illumina BeadChip data (Additional file 1: Fig. S7B).

## Discussion

Epigenetic modifications govern cellular differentiation into specific lineages and therefore DNAm is ideally suited for cellular characterization [71–73]. Previous approaches for DNAm-based deconvolution of different cell types utilized larger signatures with multiple CpGs from Illumina BeadChip datasets [5, 7, 14]. Our proof of concept study demonstrates that estimates for the cellular composition are also feasible by targeted analysis of individual CpGs. Currently, classification of cell types is often based on antibody detection of individual epitopes—thus, estimates of the cellular composition by individual CpGs may be feasible, too.

There is always a trade-off between different methods: combining a multitude of CpGs into bioinformatic predictors generally increases the precision of epigenetic signatures [74]. On the other hand, the precision of DNAm measurements at individual CpGs is higher in pyrosequencing data as compared to *β* values on Illumina BeadChips [75]. Furthermore, the choice of regimen depends on various other aspects as cost, amount of DNA, and privacy regulations (summarized in Additional file 1: Table S2): The anticipated costs for consumables may vary considerably between different countries and institutions, but they are projected to be lower for pyrosequencing than for Illumina BeadChips. It is not trivial to compare working time as this is largely dependent on the number of samples that can be processed in parallel. However, the targeted analysis with pyrosequencing is feasible within 2 to 3 days, whereas processing and analysis of Illumina BeadChips takes longer in most core facilities. The recommended amount of genomic DNA is lower for pyrosequencing (about 10–20 ng per reaction) than for Illumina BeadChips (250–500 ng DNA, albeit also feasible with less [7]). The availability of instrumentation and of bioinformatics support needs to be considered, but again these requirements are overall lower for targeted sequencing. Furthermore, regulatory requirements, such as data protection, privacy regulations, and certification of the procedures, may be easier met with targeted approaches. While we focused on pyrosequencing in this study, there are several alternative approaches for site-specific DNAm analysis, such as the Sequenom’s EpiTYPER assay, Single Base Primer Extension Assay (SNaPshot), droplet digital PCR (ddPCR), or bisulfite amplicon sequencing (BA-seq). In a recent study, we compared the accuracy of pyrosequencing, ddPCR and BA-seq for epigenetic age predictions [76]: pyrosequencing provided very robust results, albeit the PCR bias might be smaller in ddPCR. The accuracy was slightly lower for BA-seq, but this method enables longer reads with more neighboring CpGs that may be considered for pattern analysis and it can facilitate multiplexing [77]. In principle, the cell type-specific genomic regions identified in this study could also be addressed by these alternative methods for site-specific DNAm analysis. Taken together, genome scale approaches with larger libraries of CpG sites as well as targeted methods have advantages and limitations, which need to be considered.

Albeit feature selection for cell type-specific CpGs did not take biological relevance into consideration, several of them were associated with potentially functionally relevant genes. DNAm and gene expression are often correlated, but not always in the expected direction of negative for promoter CpGs and positive for gene-body CpGs [22]. Particularly, the hypomethylation in cg10673833, which is located in the promoter region of *MYO1G,* may contribute to higher expression of this gene in leukocytes. It needs to be considered that regulation of gene expression is also dependent on many other regulatory mechanisms and epigenetic modifications, such as histone code, DNA accessibility, and higher order chromatin conformation. These features may also be cell type-specific, and it is conceivable they can also be utilized for cellular deconvolution in the future.

Fibroblasts are embedded into the extracellular matrix in native tissue, but there is no distinct cell marker that allows reliable quantification of this subset. Our FibroScore was significantly increased in lung fibrosis and liver cirrhosis. Targeted pyrosequencing of the two CpGs may therefore provide a simple estimate for relative changes of fibroblasts, e.g., for staging of fibrotic diseases. Furthermore, cancer-associated fibroblasts (CAFs) play a central role for tumorigenesis, progression, and metastasis in many cancers [78, 79]. It has been shown that the fraction of CAFs, which was estimated for example by the percentage of cells that stained positive for alpha smooth muscle actin, is associated with overall survival in several types of solid cancer [80, 81]. Our findings support the notion that an epigenetic fibroblast signature can support stratification of cancer samples. In the future, it will be important to better understand the epigenetic heterogeneity of CAFs and how these signatures are affected by epigenetic aberrations of the malignant clone. While the FibroScore may be indicative of the relative fraction of fibroblasts in tissue, it does not provide a quantitative measure for the percentage of fibroblasts. To this end, we have further developed our targeted approach for deconvolution of various cell types in tissue. It is difficult to access the accuracy of our NNLS-based deconvolution for tissue samples, since we were lacking precise and validated information on their cellular composition. Nevertheless, the results of the in vitro mixes showed that deconvolution with individual cell type-specific CpGs is feasible.

A bottleneck of our analysis is the limited number of defined cellular subsets with available DNAm profiles. The lack of precise measures to distinguish between cell types is also reflected by the ongoing quest of the Human Cell Atlas Project, to define all human cell types in terms of distinctive molecular profiles and to connect this information with classical cellular descriptions (such as location and morphology) [1]. For example, fibroblasts and MSCs could possibly resemble the same type of cell [82]. On the other hand, fibroblasts are very heterogeneous and can differ greatly depending on their tissue of origin [83, 84]. For leukocyte subsets, it has been suggested that particularly cell subset-specific hypomethylation is permissive for gene expression and regulates corresponding cell functions [85]. Indeed, in our analysis, cell type-specific CpGs were predominantly hypomethylated—with the exception of iPSCs that resemble a rather non-differentiated ground state. In previous work, we have extensively studied characteristic DNAm patterns of hematopoietic subsets [16], but we have chosen to not over-represent the hematopoietic compartment in our deconvolution approach and to therefore combine all leukocytes into one category. Our hypomethylated CpGs for leukocytes, fibroblasts, endothelial cells, epithelial cells, hepatocytes, glia, neurons, and iPSCs cannot span the many facets of cellular classification but at least most cell types can be subordinated to at least one of these broad categories.

## Conclusions

Our results demonstrate that individual CpGs, which are particularly hypomethylated in specific cell types, can be used to estimate the fraction of fibroblasts or the composition of cellular mixes and tissues. In contrast to genome-wide DNAm profiles, targeted analysis, e.g., by pyrosequencing, provides new perspectives for small amounts of DNA and to derive robust procedures according to directives for in vitro diagnostic devices. Such analysis may be useful to gain insight into the composition of unknown tissue specimen or to correlate the percentage of specific cellular subsets with clinical parameters. Furthermore, it might provide estimates for the composition of cell-free DNA (cfDNA), which is increasingly relevant for liquid biopsy [7, 86].

## Methods

### Data acquisition and processing of DNAm profiles

We compiled a curated dataset of DNAm profiles (450K and EPIC Illumina BeadChip platforms) of well-characterized and non-malignant human cell types. All analysis and data retrieval was performed with the R programming language v3.6.2 and functions from Bioconductor v3.9. The data was retrieved from Gene Expression Omnibus (through the GEOquery v2.52.0 R package (GEOquery, RRID:SCR_000146), Additional file 1: Table S1), and data processing was performed using the minfi v1.30.0 R package (minfi, RRID:SCR_012830) and in-house scripts. Features were limited to CpGs shared between the 450K and EPIC platforms, and we excluded probes related to sexual chromosomes and probes not shared across all samples (missing data), resulting in 415,366 CpGs for further selection. During the data acquisition process (at time of analysis), several samples and features were dropped due to conflicting or missing names, bad file formatting, and missing data. In the end, we had a total of 579 samples from 14 different cell types. For samples where raw data was available (IDAT files), ssNoob normalization method was applied [87]; otherwise, no additional normalization of beta values was performed. To avoid bias and overfitting, samples were divided into two independent datasets, a training (*n* = 409) and validation set (*n* = 170). Datasets from The Cancer Genome Atlas (TCGA) project (level 1 methylation array data) were downloaded and preprocessed with the TCGAbiolinks v2.12.6 (TCGAbiolinks, RRID:SCR_017683) and SeSAMe v1.2.0 packages in R (except for TCGA-STAD, which at the time of the analysis was unreachable) following their respective pipelines (Fig. 2; Additional file 1: Fig. S2) [88, 89].

Gene expression data (Affymetrix UG 133 Plus 2.0) from corresponding genes of the selected cell type-specific CpGs was extracted from www.biogps.org and the Primary Cell Atlas dataset [67]. Groups have been adjusted to fit the selected cell types. Redundant samples (e.g., time course experiments), experimentally treated samples (e.g., drugs, antibodies), tissue samples, and cells differentiated from iPSC or ES cells have been removed from the dataset (from a total of 754 samples, down to 383). If different probe sets were available for one gene, we selected the one that addressed the entire gene and correlated best with gene expression.

### Feature selection and signatures for classification and deconvolution

In order to find the best CpGs to perform classification, we subjected the training data to a stratified k-fold cross-validation setup (*k* = 10). We defined *C*_*i*_ as our cell of interest and *C*_*other*_ as the class that englobes all the other cell types. For a given fold, we calculate the difference in means between *C*_*i*_ and *C*_*other*_ (dMean) and the sum of variances within *C*_i_ and *C*_other_ (sVar) for each CpG. The relationship between mean and variances is exemplified in Fig. 1b, where we assume that CpGs with higher absolute dMean, and lower sVar were considered more discriminative. To capture a set of discriminative CpGs, we define a parabola function and select all CpGs for which *y < (ax)*^*2*^ (Fig. 1b). Initially, *a* is set to 0.1. If less than 10 hypermethylated (*x* > 0) or 10 hypomethylated (*x* < 0) CpGs are selected, *a* is incremented by 0.1 until the previous criteria is reached. Next, we compute the area under the precision-recall curve (AUPR) on the remaining folds and scale it by the absolute dMean [90]. We consider here hypo- and hypermethylated CpGs separately for estimating dMean. The above procedure is repeated for each fold. A final score is obtained by the average scaled AUPR multiplied by the proportion of folds where a CpG was selected as a top scoring candidate. This is then used to obtain a final CpG ranking. This measure selects CpGs present in more folds, having higher AUPR and higher absolute dMean. The best iPSC CpG was not suitable for primer design for pyrosequencing and therefore the second best was selected for this cell type. For the FibroScore, we used the F1-score (without scaling) and selected from the best CpGs, one hypo- and one hypermethylated CpG, after initial screening with pyrosequencing.

### Deconvolution of cell type proportions

Using the cell type-specific CpGs previously selected for classification and their mean methylation value (for each cell type) on the training dataset as our reference matrix, we applied a reference-based non-negative least-squares (NNLS) algorithm [16, 69]. An application for cell type deconvolution is provided as a separate Excel tool (Additional file 2) and as the DeconvolutionApp, https://costalab.ukaachen.de/shiny/tmaie/deconapp/ (accessed 24 July 2020) [91].

### Survival analysis

A multivariate survival analysis was performed on TCGA data using Cox proportional hazards models, taking into account (when available) sex, age, tumor stage, and the FibroScore stratified by median. Plots were created with the survival v3.1.12 and survminer v0.4.6 packages in R [92, 93]. *P* values are based on the log-rank test.

### Cell culture

Human mesenchymal stromal cells [94], dermal fibroblasts [84], human umbilical vein endothelial cells (HUVECs) [38], and iPSCs [94, 95] were isolated and thoroughly characterized as described in our previous work. Human cell lines HepG2, HuH-7, Hep3B, and HaCat were maintained at RWTH Aachen Medical School under standard culture for isolation of genomic DNA. For HepG2 and HaCat, DNA was directly isolated from cryopreserved vials.

### Isolation of genomic DNA and bisulfite conversion

Genomic DNA from cells and tissues was isolated with the NucleoSpin® Tissue Kit (Macherey-Nagel) and from blood (150 μl) with the QIAamp DNA Blood Mini Kit (Qiagen). DNA concentration was measured using the NanoDrop™ 2000 spectrophotometer (Thermo Scientific™) and bisulfite converted using the EZ DNA Methylation Kit (Zymo Research).

### Pyrosequencing

Bisulfite converted DNA (10–20 ng) was amplified with a region-specific biotinylated/unmodified DNA primer pair (Metabion; Additional file 1: Table S3) using the PyroMark PCR Kit (Qiagen) according to the manufactures instructions: Initial activation at 95 °C for 15 min, then 45 cycles of 30 s at 94 °C, 30 s at 56 °C, and 30 s at 72 °C followed by a final extension at 72 °C for 10 min. Pyrosequencing was performed on the PyroMark Q96 and the Q48 Autoprep platforms. Exemplary pyrograms are provided in Additional file 1: Fig. S8. The assay for the neuron-specific CpG site was designed for the complementary strand to stay within a more reasonable sequencing distance. The results were analyzed using the Pyro Q-CpG 1.0.9 or the PyroMark Q48 Advanced Software, respectively.

### Quantification and statistical analysis

In total, we used DNAm profiles of 579 samples from 46 different studies for training and validation sets. The pyrosequencing signatures were validated with four cell lines, 12 MSC samples, 6 fibroblast samples, 4 HUVEC preparations, 5 iPSC lines, 8 blood samples, and 14 different tissue samples. To estimate the significance of differential DNAm and FibroScore in the lung fibrosis and liver cirrhosis datasets, we utilized the two-sided *t* test: *** < 0.001, ** < 0.01, * < 0.05. *P* values for overall survival in cancer are based on the log-rank test.

## Supplementary Information

**Additional file 1: Figure S1.** Selection of cell-type specific CpGs for fibroblasts. **Figure S2.** Cox proportional hazards adjusted survival curves for FibroScore. **Figure S3.** Survival analysis in other types of cancer. **Figure S4.** Genomic context of cell-type-specific CpG sites. **Figure S5.** Gene expression of CpG related genes. **Figure S6.** Comparison of different deconvolution methods. **Figure S7.** Deconvolution of cell mixtures based on individual cell-type-specific CpGs. **Figure S8.** Representative pyrograms. **Table S1.** 450k/EPIC Illumina BeadChip datasets used in this study. **Table S2.** Advantages and limitations of pyrosequencing versus Illumina BeadChips. Table S3. Primer DNA sequences used for pyrosequencing.

**Additional file 2.** Excel application for cell type deconvolution. An application for cell type deconvolution is provided as separate Excel tool. The mean DNAm values for the cell-type-specific CpGs of the Illumina BeadChip training dataset are given as reference matrix. Furthermore, the application allows NNLS predictions to estimate the cellular composition in independent datasets. This table was generated in analogy to the NNLS application for Epi-Blood-Count [16].

## Data Availability

The datasets analyzed during the current study are available in the Gene Expression Omnibus (GEO): GSE34486, GSE40699, GSE41933, GSE43976, GSE50222, GSE52025, GSE52112, GSE58622, GSE59065, GSE59091, GSE59250, GSE59796, GSE60753, GSE63409, GSE65078, GSE68134, GSE71955, GSE74877, GSE77135, GSE79144, GSE79695, GSE82234, GSE85647, GSE87095, GSE87177, GSE88824, GSE92843, GSE95096, GSE98203, GSE99716, GSE103253, GSE107226, GSE51921, GSE53302, GSE68851, GSE71244, GSE74486, GSE85566, GSE86258, GSE86829, GSE87797, GSE104287, GSE106099, GSE109042, GSE111396, GSE122126, GSE41826, GSE60753, GSE63704, and The Cancer Genome Atlas (TCGA) repositories (see also Additional file 1: Table S1). A DeconvolutionApp is provided at https://costalab.ukaachen.de/shiny/tmaie/deconapp/ (accessed 24 July 2020) [91].
