# Supplementary material for: Deconvolution of cellular subsets in human tissue based on targeted DNA methylation analysis at individual CpG sites
Source: BMC Biol. 2020 Nov 24;18:178. doi: 10.1186/s12915-020-00910-4 (PMC7687708; doi:10.1186/s12915-020-00910-4)
Supplement: Supplementary file 1 — Additional file 1: Figure S1. Selection of cell-type specific CpGs for fibroblasts. Figure S2. Cox proportional hazards adjusted survival curves for FibroScore. Figure S3. Survival analysis in other types of cancer. Figure S4. Genomic context of cell-type-specific CpG sites. Figure S5. Gene expression of CpG related genes. Figure S6. Comparison of different deconvolution methods. Figure S7. Deconvolution of cell mixtures based on individual cell-type-specific CpGs. Figure S8. Representative pyrograms. Table S1. 450k/EPIC Illumina BeadChip datasets used in this study. Table S2. Advantages and limitations of pyrosequencing versus Illumina BeadChips. Table S3. Primer DNA sequences used for pyrosequencing. [file 12915_2020_910_MOESM1_ESM.pdf]

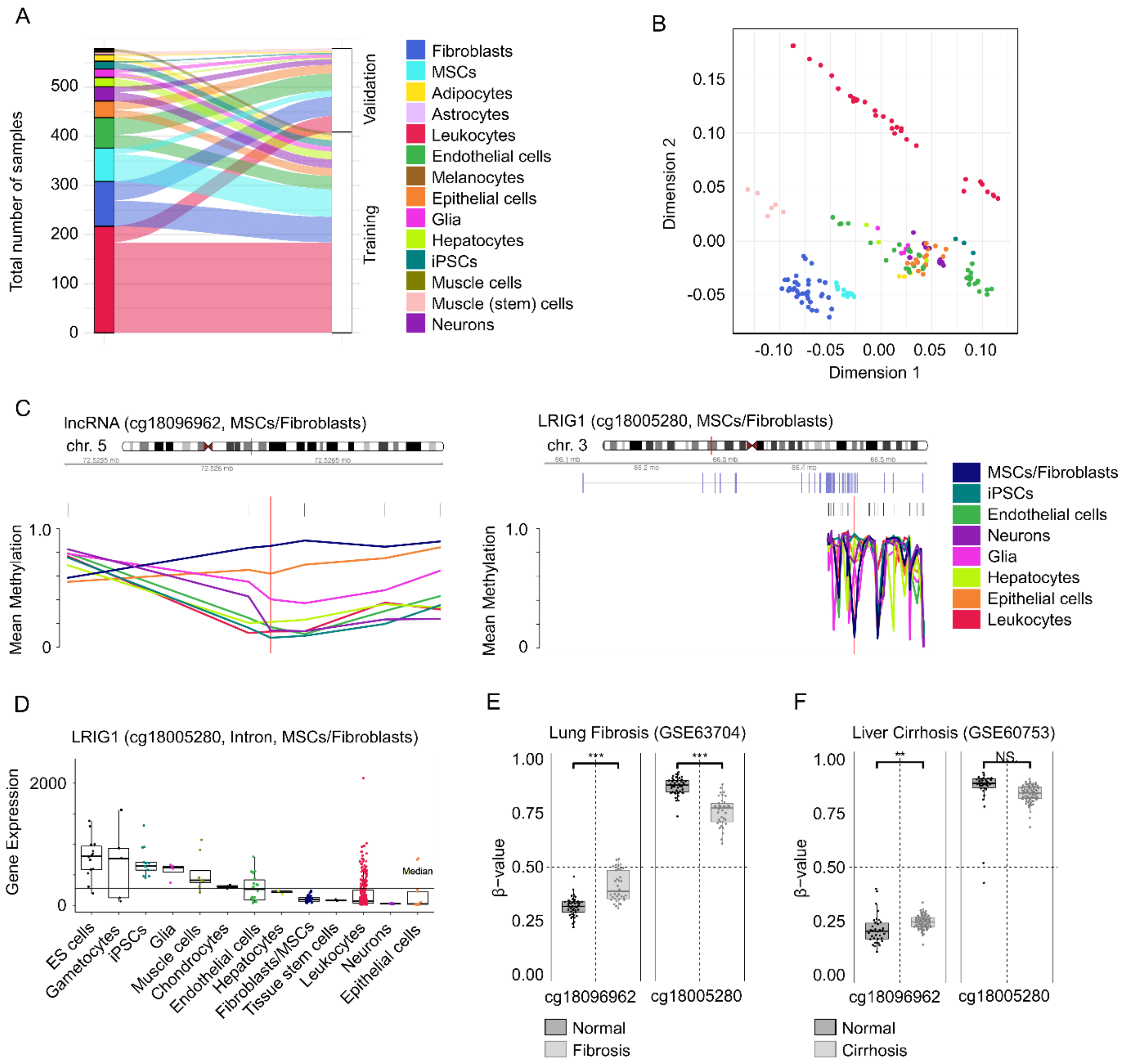

Supplemental Figure S1: Selection of cell-type specific CpGs for fibroblasts

(A) Split of total samples (n = 579) into training (n = 409) and validation set (n = 170).

(B) Multidimensional scaling (MDS) plot of the validation data (n = 170) shows that samples cluster by cell type. For the analysis, all CpGs shared between the 450K and the EPIC BeadChip were included (except XY chromosomes).

(C) Chromosomal location and association with corresponding genes for the selected fibroblast-specific CpGs: lncRNA (*RP11-60A8.1*) and leucine rich repeats and immunoglobulin like domains 1 (*LRIG1*). Shown are mean β values from the training data set of neighboring CpGs for the respective cell types. The red lines depict the relevant CpGs.

(D) Gene expression estimates of the Primary Cell Atlas (67) for *LRIG1* (Affymetrix UG 133; probe 211596_s_at). Gene expression is relatively low in fibroblasts. Thus, there was no evidence for cell-type specific gene expression. Gene expression data was not available for the lncRNA RP11-60A8.1.

(E) DNAm levels (β values) of the two selected CpGs from the FibroScore in the lung fibrosis dataset GSE63704 (450K BeadChip) (63). Two-sided t-test: *** $p < 0.001$.

(F) DNAm levels (β values) of the two selected CpGs from the FibroScore in the liver cirrhosis dataset GSE60753 (450K BeadChip) (29). Two-sided t-test: : ** $p < 0.01$, NS = not significant.

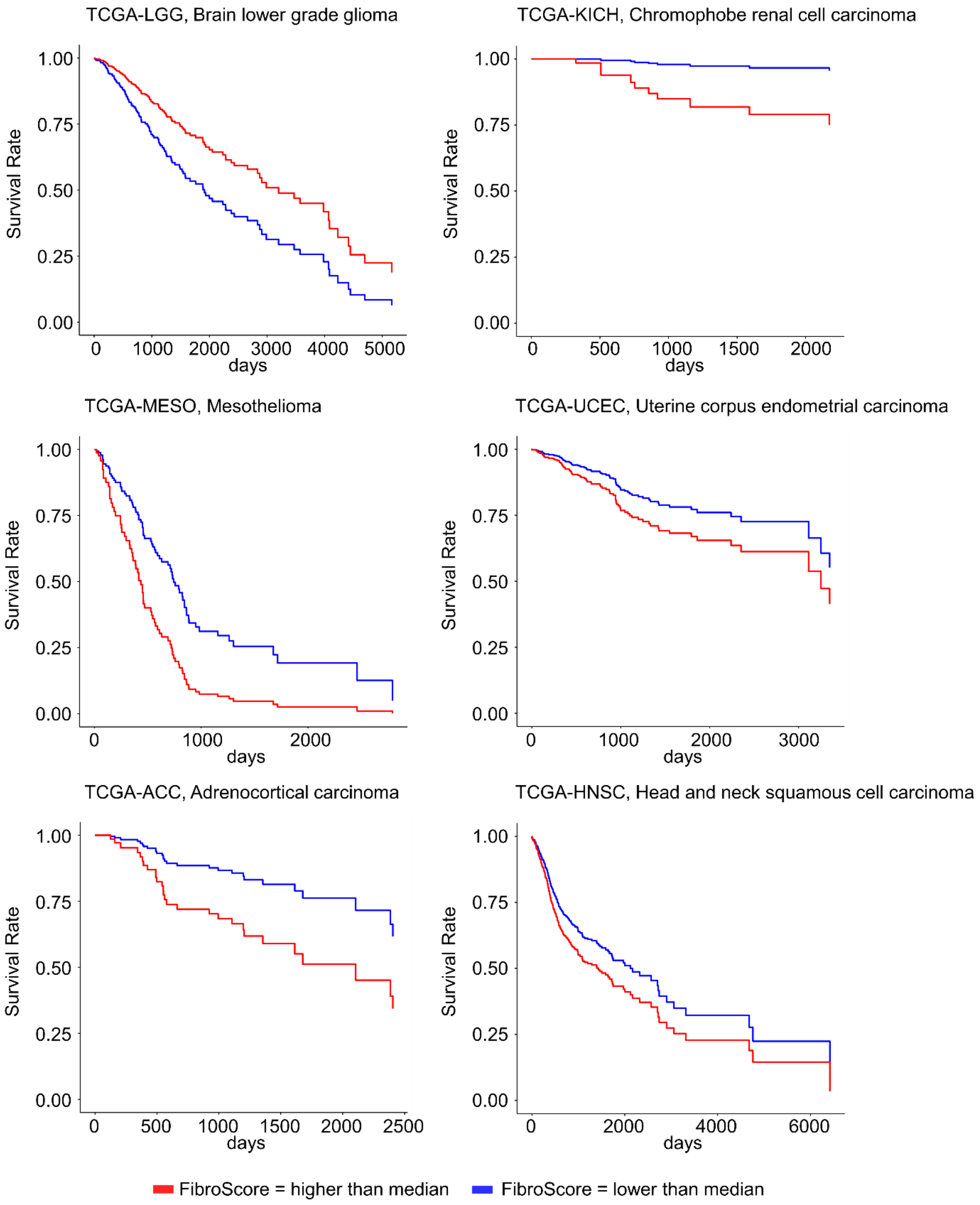

Supplemental Figure S2: Cox proportional hazards adjusted survival curves for FibroScore

Survival curves for the six types of cancer for which there is a significant difference in overall survival for patients with either high or low FibroScore. Depicted is the difference between the groups stratified as low or high FibroScore. The model takes (when available) sex, age, tumor stage and FibroScore into account.

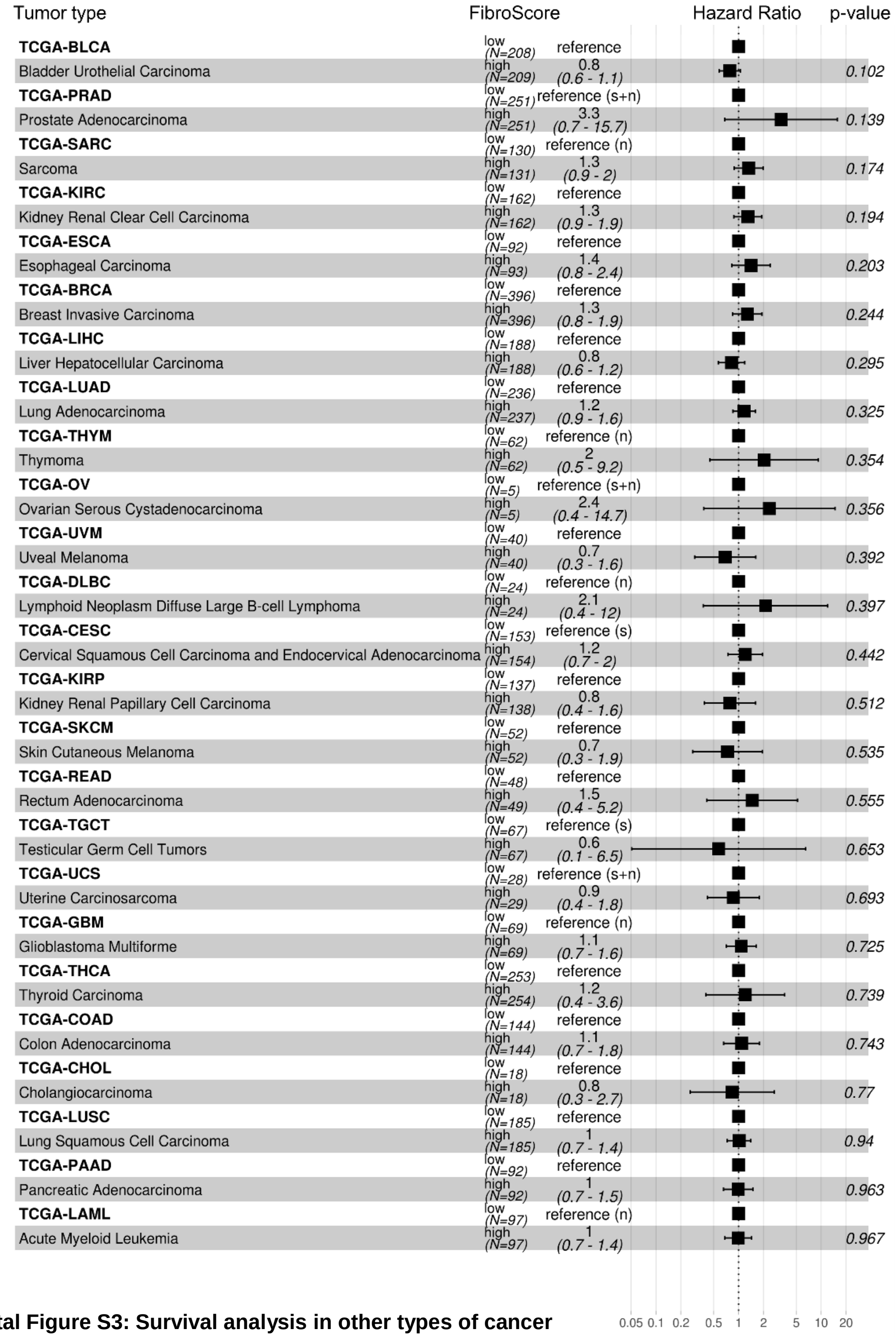

Supplemental Figure S3: Survival analysis in other types of cancer

Hazards ratios from Cox proportional hazards models for datasets from The Cancer Genome Atlas (TCGA). Depicted are all types of cancer for which there is no significant difference in overall survival for patients with either high or low FibroScore. Unless specified otherwise, models take into account sex, age, tumor stage, and the FibroScore stratified by the median (450K BeadChip data). If some of these parameters were not available, we indicated missing cofactors next to the reference: s = sex, a = age, and n = stage.

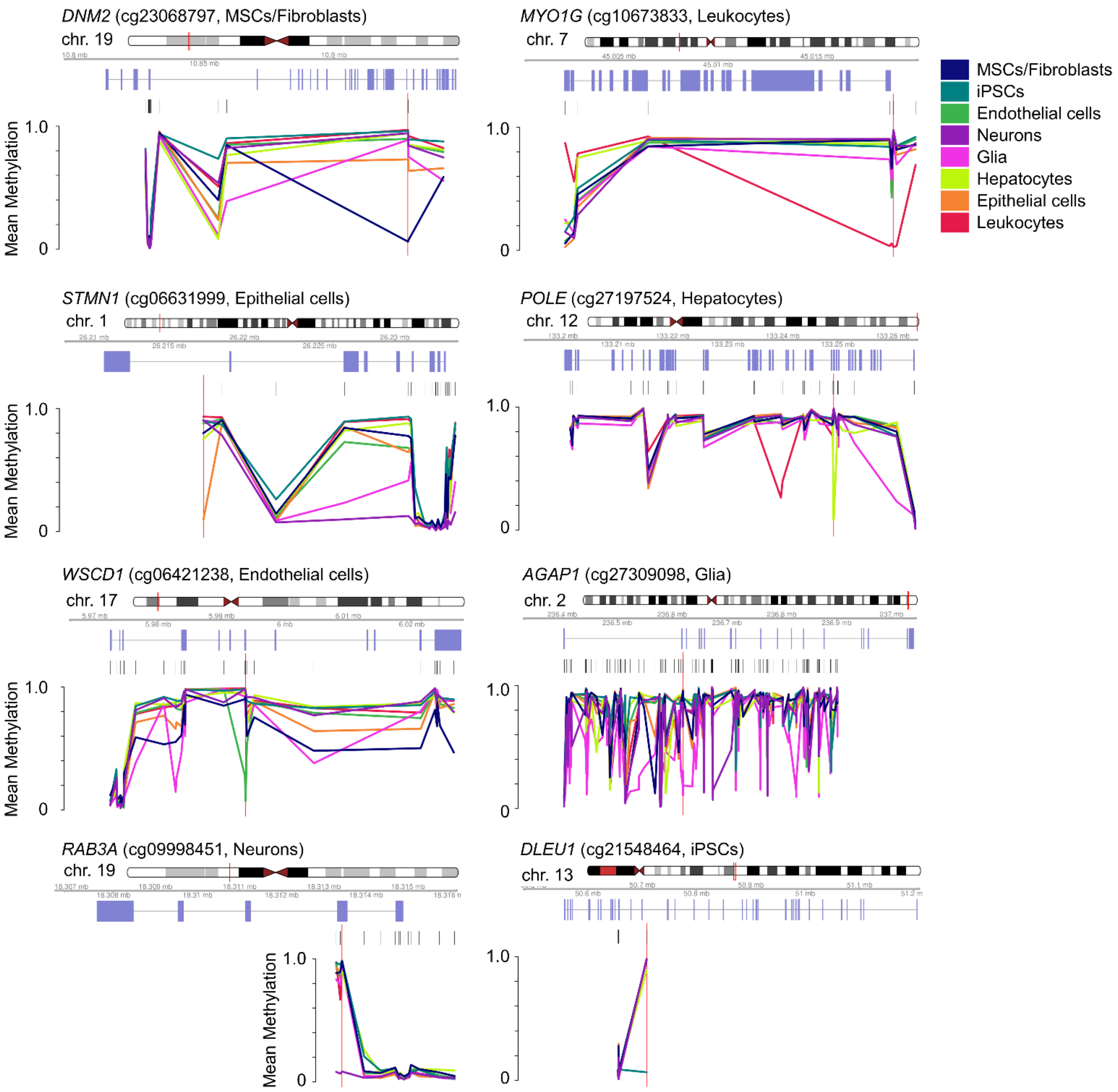

Supplemental Figure S4: Genomic context of cell-type-specific CpG sites

Chromosomal location and association with corresponding genes for the selected cell-type-specific CpGs: Dynamin 2 (*DNM2*), Myosin IG (*MYO1G*) encodes for the human minor histocompatibility antigen HA-2, which is only expressed in hematopoietic cells (65), Stathmin 1 (*STMN1*), DNA polymerase epsilon catalytic subunit A (*POLE*), WSC Domain Containing 1 (*WSCD1*), ArfGAP With GTPase Domain, Ankyrin Repeat And PH Domain 1 (*AGAP1*) is associated with neurodevelopmental disorders and overexpressed in brain (66), RAS-Associated Protein RAB3A (*RAB3A*) is also highly expressed in brain (66), and the lncRNA Deleted In Lymphocytic Leukemia 1 *(DLEU1)*. Shown are mean β values from the training data set of neighboring CpGs for the respective cell types. The red lines depict the relevant CpGs.

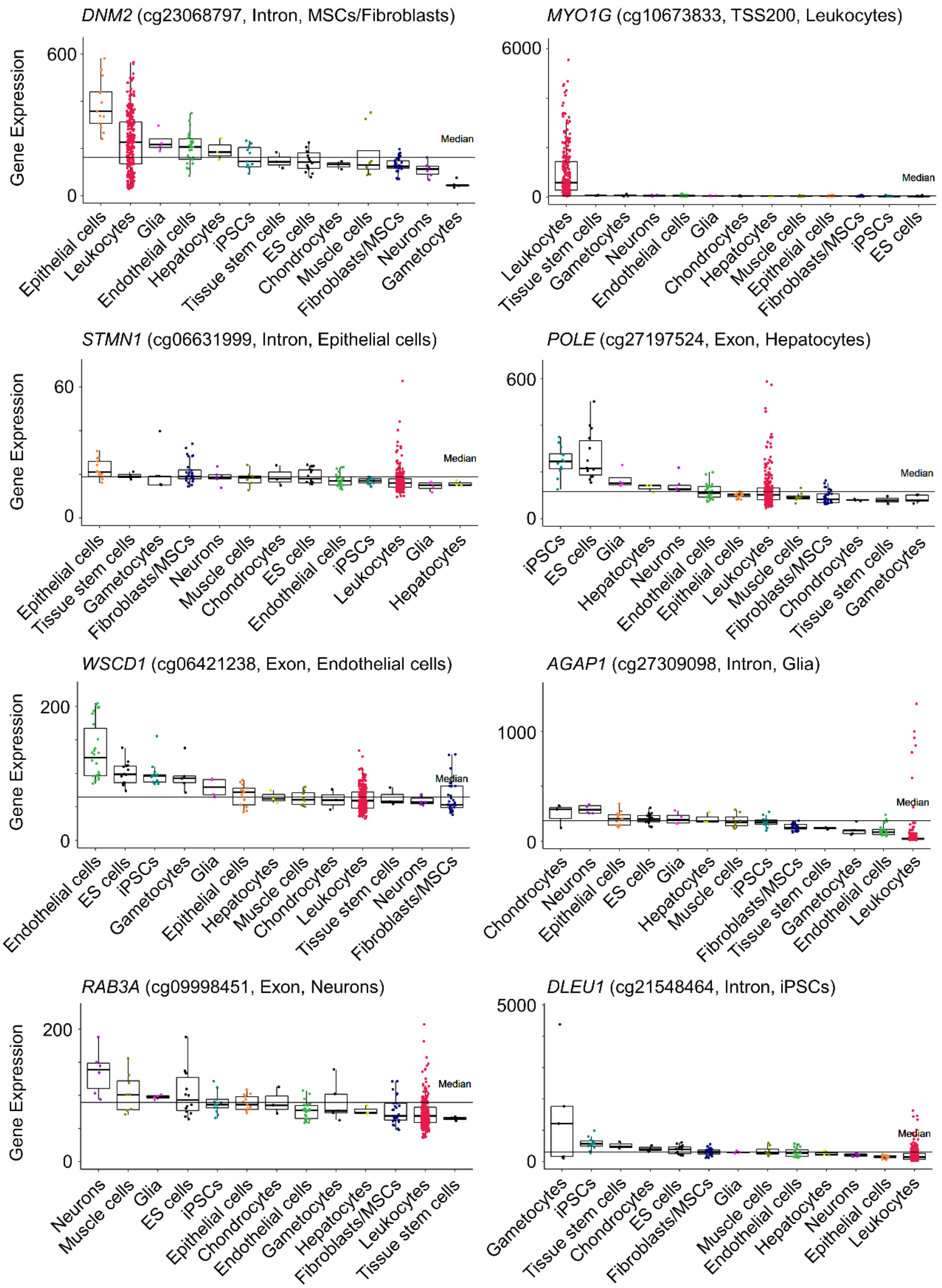

Supplemental Figure S5: Gene expression of CpG related genes

Gene expression (Affymetrix UG 133 Plus 2.0) of corresponding genes for the selected cell-type-specific CpGs: *DNM2* (202253_s_at), *MYO1G* (244654_at), *STMN1* (1552803_a_at), *POLE* (216026_s_at), *WSCD1* (213157_s_at), *AGAP1* (204066_s_at), *RAB3A* (204974_at) and the lncRNA *DLEU1* (219076_s_at) from the Primary Cell Atlas (67).

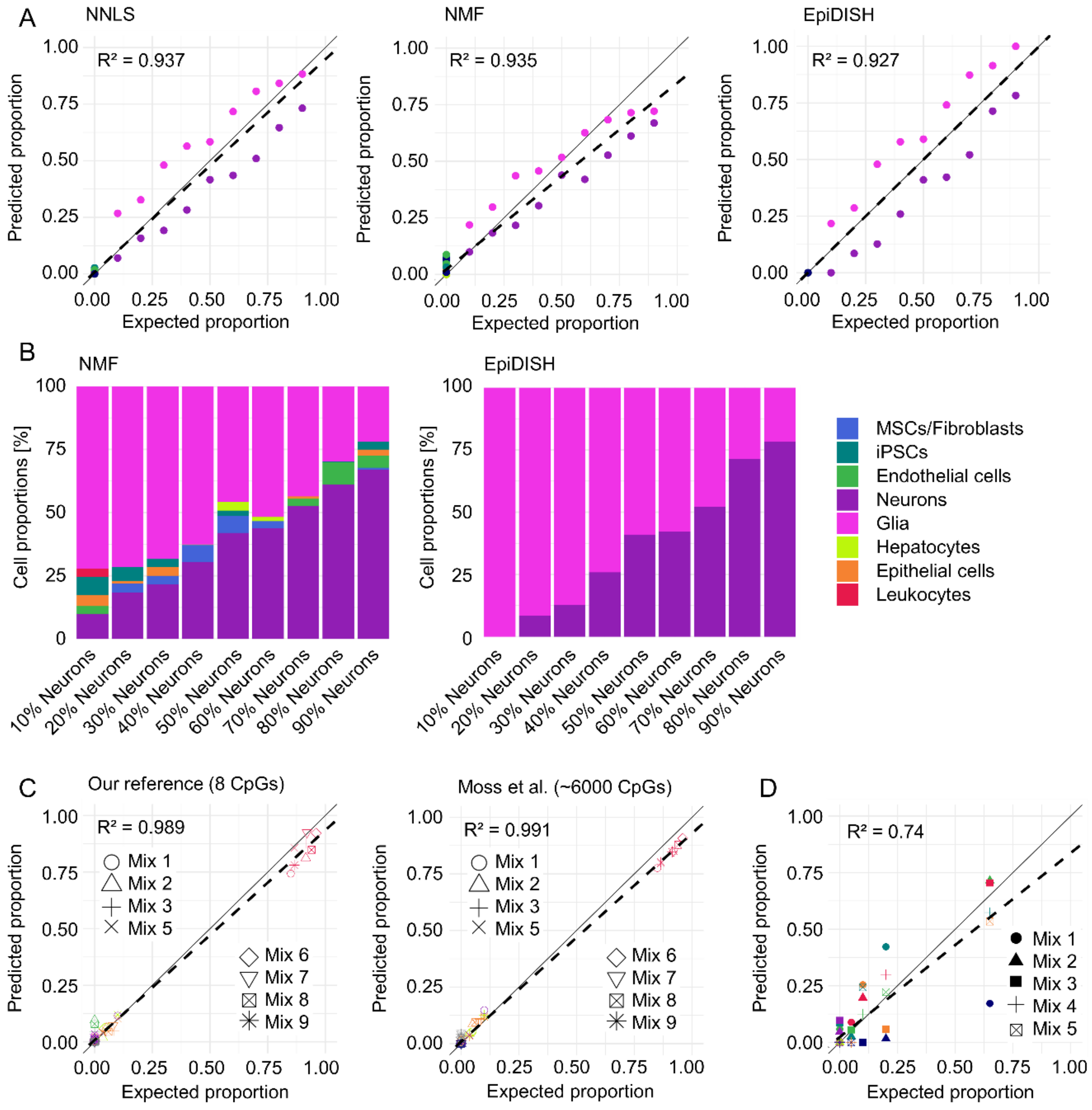

Supplemental Figure S6: Comparison of different deconvolution methods

(A) In addition to the NNLS model, two alternative deconvolution approaches were considered for *in vitro* neuron-glia-DNA mixes from dataset GSE41826 (68): non-negative matrix factorization (NMF) and EpiDISH (69, 70). Comparison of expected *versus* predicted DNA proportions by deconvolution with different algorithms (NNLS, NMF and EpiDISH).

(B) The predicted cell fractions for the neuron-glia-DNA mixes from dataset GSE41826 (68) are depicted based on the eight cell-type-specific CpGs for NMF and EpiDISH. The best correlation of the predicted cell fractions with the real mixture of neurons/glia was observed for the NNLS-based deconvolution (Figure 4), while EpiDISH did not misclassify any other cell type.

(C) Cell type DNA mixes from dataset GSE122126 (7). Comparison of expected *versus* predicted DNA proportions by deconvolution with different reference matrices.

(D) Pyrosequencing of DNA mixes. Comparison of expected *versus* predicted DNA proportions by deconvolution.

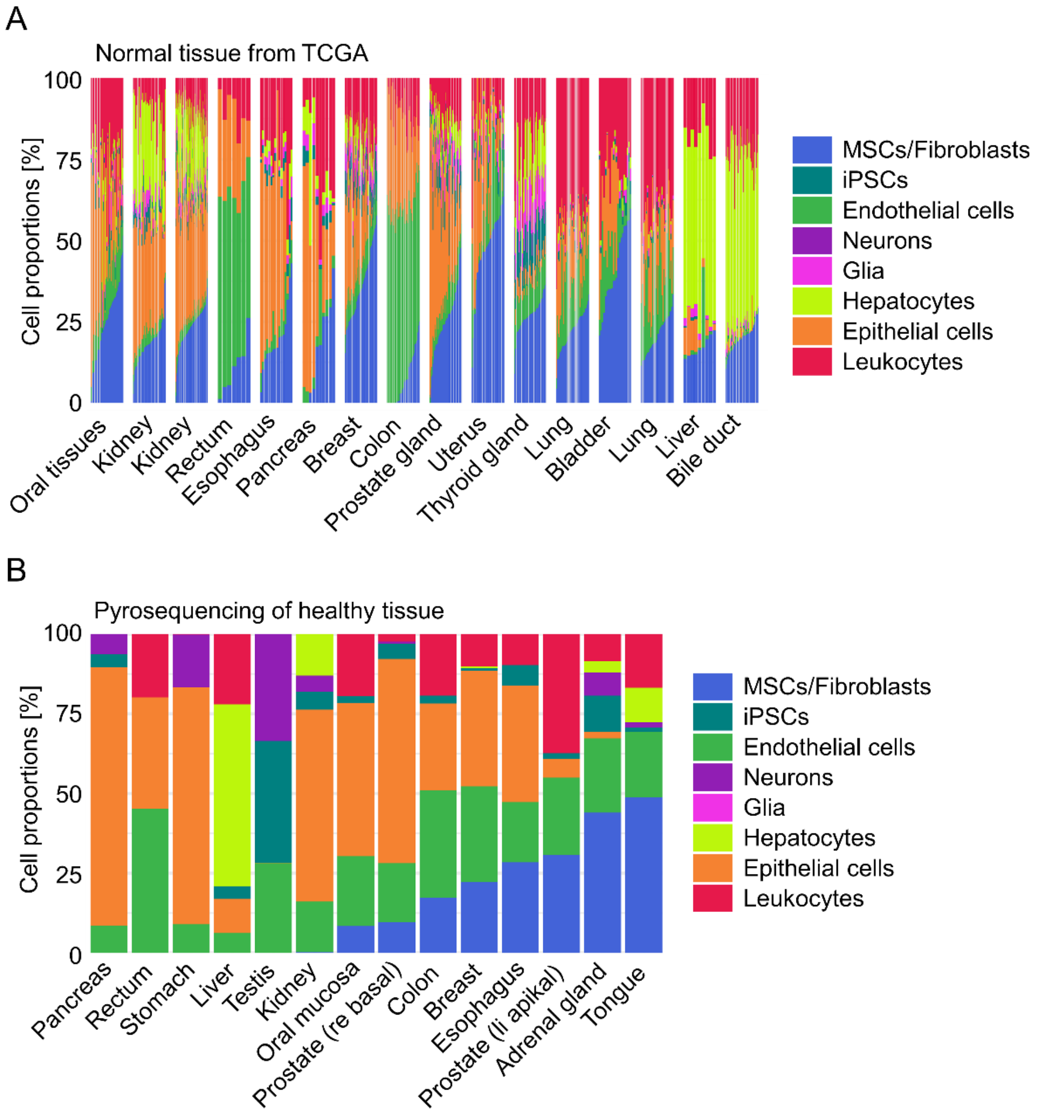

Supplemental Figure S7: Deconvolution of cell mixtures based on individual cell-type-specific CpGs

(A) Deconvolution of normal tissues from the TCGA database (450K BeadChip). Shown are estimated cellular fractions. The relevant CpG for neurons was not available for TCGA datasets and therefore left out.

(B) Deconvolution of healthy tissues based on pyrosequencing of DNAm at the eight relevant CpGs.

cg18096962 (MSCs/Fibroblasts)

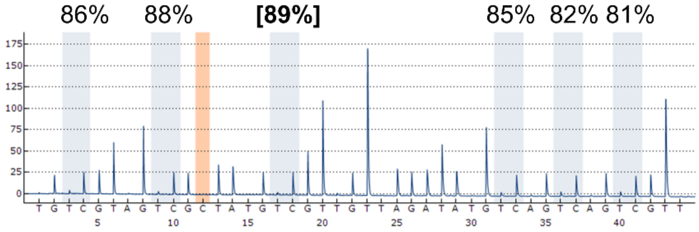

Sequence to analyze:
GYGTTGGGYGTAGYGGTTTTGTTTTTTTAGATT
AGGGYGYGYGTTTTTGG

Specific for: MSCs/Fibroblasts
Methylation in target cell type: hypermethylated
Sample: MSCs

cg18005280 (MSCs/Fibroblasts)

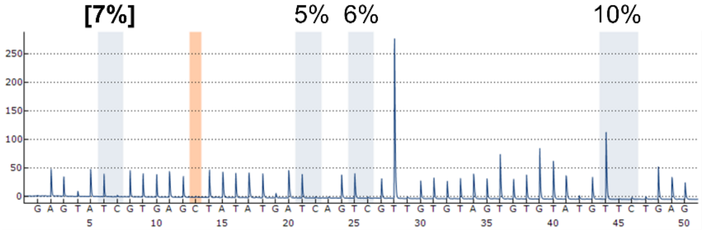

Sequence to analyze:
AGAYGTGAGTATATAYGYGTTTTTTTTTGTGTAG
TTGTGGGTTAGTTTYGGAGT

Specific for: MSCs/Fibroblasts
Methylation in target cell type: hypomethylated
Sample: MSCs

cg23068797 (MSCs/Fibroblasts)

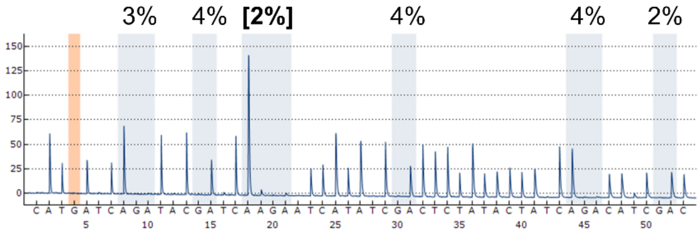

Sequence to analyze:
AATACRATTCCRCCRAAAAATCAATAACCRCCT
TCCTAATACTACCRACACRCA

Specific for: MSCs/Fibroblasts
Methylation in target cell type: hypomethylated
Sample: MSCs

cg10673833 (Leukocytes)

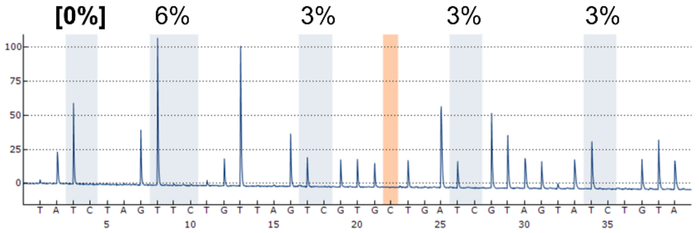

Sequence to analyze:
ATTYGGTTTTTYGTTTTTGGYGTGTAAAYGGGT
TAGATYGTTAGG

Specific for: Leukocytes
Methylation in target cell type: hypomethylated
Sample: Whole blood

cg06631999 (Epithelial cells)

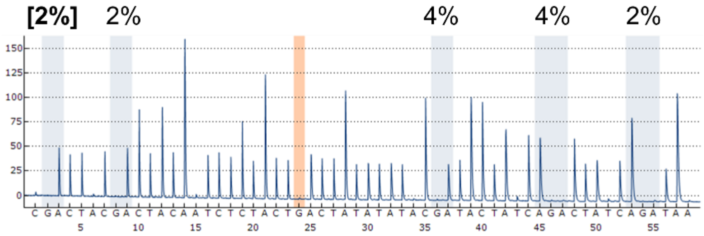

Sequence to analyze:
RCTCRCCTAACAAAATCTCCTAAACTACTAAAT
ATATCCCRTAAACCCTAACCRACCTACRAATAA
AAT
Specific for: Epithelial cells
Methylation in target cell type: hypomethylated
Sample: Keratinocytes

cg27197524 (Hepatocytes)

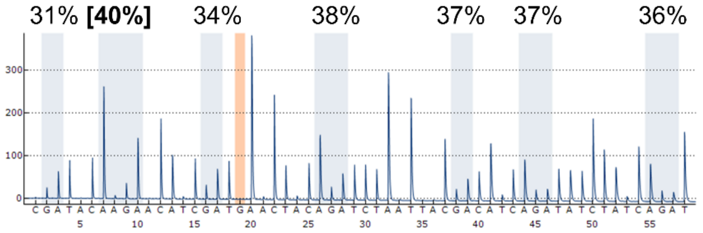

Sequence to analyze:
RTCRAAAACCACRTAAAAACCCTCRAATCTAAA
ATTTTCCRCAACRATATCCCTTACCRATTTAAAA

Specific for: Hepatocytes
Methylation in target cell type: hypomethylated
Sample: Liver

cg06421238 (Endothelial cells)

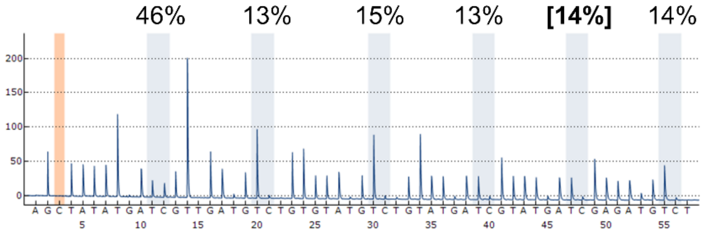

Sequence to analyze:
GGTATATTTAYGTTTTTTGGAGTTYGGTTGTAG
TTYGTTTATAYGGTATAYGGAGAGTYG

Specific for: Endothelial cells
Methylation in target cell type: hypomethylated
Sample: HUVECs

cg27309098 (Glia)

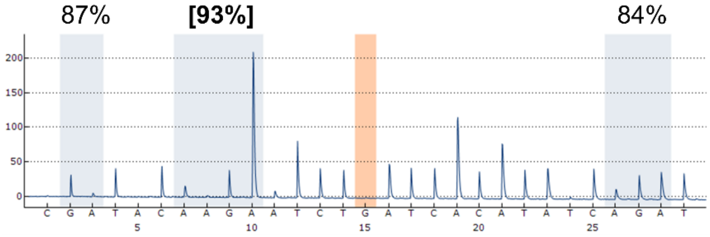

Sequence to analyze:
RTCRAAAAAATTCTATCAAACAATACRATA

Specific for: Glia
Methylation in target cell type: hypomethylated
Sample: HUVECs (as negative example)

cg09998451 (Neurons)

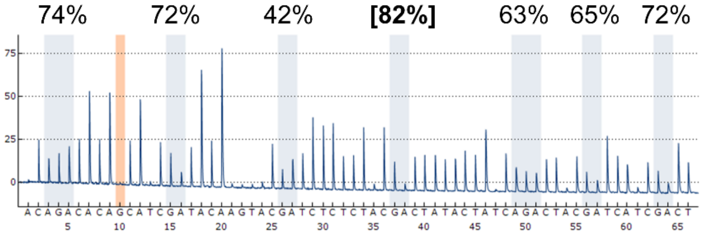

Sequence to analyze:
CRACAACAACAACRTAAACAAAACRTCCTTCC
TCTTCCRCTATACTAACRACTCRTTCACRCCTA

Specific for: Neurons
Methylation in target cell type: hypomethylated
Sample: MSCs (as negative example)

cg21548464 (iPSCs)

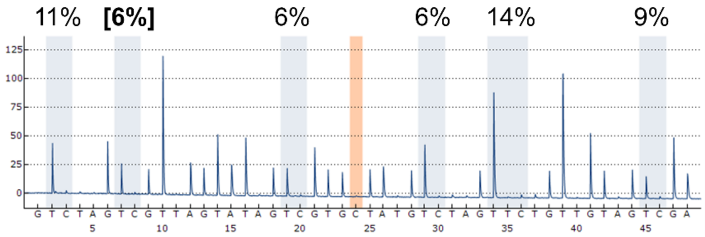

Sequence to analyze:
TYGGYGTTTTTAGTTATTGYGGTGTAGTYGTTT
TYGTTTTTTGGGTGYGGGAT

Specific for: iPSCs
Methylation in target cell type: hypomethylated
Sample: iPSCs

Supplemental Figure S8: Representative pyrograms

Representative pyrograms are depicted for each pyrosequencing assay. Bisulfite conversion controls are marked in orange, CpG sites are marked in blue and the methylation level of the target CpG site is in square brackets. Images have been exported from the PyroMark Q48 Autoprep software from Qiagen.

Supplemental Table S1. 450k/EPIC Illumina BeadChip datasets used in this study

GSE	SAMPLE TYPE	NUMBER OF SAMPLES	REFERENCE IN MAIN TEXT
**Training dataset**			
GSE34486*	endothelial cells	10	17
GSE40699	muscle cells, epithelial cells, hepatocytes, fibroblasts, astrocyte, endothelial cells	26	18
GSE41933	mesenchymal stromal cells	12	19
GSE43976	leukocytes	10	20
GSE50222	leukocytes	8	21
GSE52025	fibroblasts	2	22
GSE52112	mesenchymal stromal cells	34	23
GSE58622	adipocytes	10	24
GSE59065	leukocytes	20	25
GSE59091	induced pluripotent stem cells, fibroblasts	12	26
GSE59250	leukocytes	60	27
GSE59796	leukocytes	4	28
GSE60753	hepatocytes	15	29
GSE63409	leukocytes	30	30
GSE65078	induced pluripotent stem cells, fibroblasts	8	31
GSE68134	induced pluripotent stem cells, fibroblasts	6	32
GSE71955	leukocytes	20	33
GSE74877	epithelial cells, melanocytes, mesenchymal stromal cells, fibroblasts, endothelial cells	9	34
GSE77135	fibroblasts	20	35
GSE79144	glia, neurons	18	36
GSE79695	mesenchymal stromal cells	12	37
GSE82234	endothelial cells	3	38
GSE85647	leukocytes	6	39
GSE87095	leukocytes	10	30
GSE87177	endothelial cells	1	41
GSE88824	leukocytes	16	42
GSE92843	epithelial cells	1	43
GSE95096	fibroblasts	1	44
GSE98203	neurons	10	45
GSE99716	endothelial cells	1	46
GSE103253*	endothelial cells	10	47
GSE107226	fibroblasts	4	48
**Validation dataset**			
GSE34486*	endothelial cells	6	17
GSE51921	iPSCs, fibroblasts	6	49
GSE53302	muscle stem cells	6	50
GSE68851	fibroblasts	12	51
GSE71244	leukocytes	24	52
GSE74486	glia, neurons	15	53
GSE85566	epithelial cells	6	54
GSE86258	fibroblasts	7	55
GSE86829	fibroblasts	3	56
GSE87797	mesenchymal stromal cells	12	57
GSE103253*	endothelial cells	15	47
GSE104287	leukocytes	8	58
GSE106099	feto-placental endothelial cells	12	59
GSE109042	buccal epithelial cells	6	60
GSE111396	fibroblasts	14	61
GSE122126	adipocytes, neurons, hepatocytes, endothelial cells, epithelial cells, leukocytes	18 (13 EPIC)	7
**Other datasets**			
GSE41826	glia-neuron DNA mixes	9	68
GSE60753	normal and cirrhotic liver	100	29
GSE63704	normal and fibrotic lung	80	63
GSE122126	cell type DNA mixes	8	7

* Samples from these studies were considered either for training or validation sets

Supplemental Table S2. Advantages and limitations of pyrosequencing *versus* Illumina BeadChips

	PYROSEQUENCING OF 8 CPGS	ILLUMINA METHYLATIONEPIC BEADCHIP (~ 850.000 CPGS)
**Consumables per sample (including bisulfite conversion)**	~ 40 €	175 € to 600 € *
**Working time**	10 h **	Usually performed by service provider or core facility
**Time until results**	2-3 days	weeks/months (dependent on service provider)
**Preferred total DNA amount**	200 - 500 ng	1200 ng
**Minimum input quantity of converted DNA**	80 - 160 ng (10-20 ng per reaction)	250 ng
**Requirement of bioinformatic skills**	lower	higher
**Data protection and privacy regulations**	analysis in house, no genome wide data	Genome wide profiles may theoretically reveal donor identity

* The given price range comprises consumables and personnel costs. On our requests, there was a considerable price range between different providers and with possible cooperation agreements. Consumable costs only for Infinium MethylationEPIC BeadChip Kit are provided by Illumina about 250€/sample.

** The working time needs to be taken into account for cost calculation. However, this is largely dependent on the number of samples that can be processed in parallel.

Supplemental Table S3. Primer DNA sequences used for pyrosequencing

NAME	DNA SEQUENCE
cg18096962_Forward	GAGTATTGGGTTTATTTAGTTTTAGGAT
cg18096962_Reverse_Biotin	TCAAATTCTATTTACTACCCTCTTCC
cg18096962_Sequencing	GTTTTTATTTTTGAG
cg18005280_Forward	TATTGGTGTTATTGGGGGAGG
cg18005280_Reverse_Biotin	CCCACAACCATTCTAAAACAATC
cg18005280_Sequencing	TGTTATTGGGGGAGG
cg10673833_Forward	TGTTGTTAGGGTTGGAAGTTAATTT
cg10673833_Reverse_Biotin	CACCAACCTCCTCCAATACTAATATAA
cg10673833_Sequencing	GGGGAGGATTTAGT
cg06421238_Forward	TTGTGGGGGATGGGTAGT
cg06421238_Reverse_Biotin	ACCTCCTCCCTACAAATCCTATATCT
cg06421238_Sequencing	GATAAAGTTTAGGAAGAGGTT
cg06631999_Forward_Biotin	GGGTTGTTTTTTGGTTATTAGAGTTAGGTA
cg06631999_Reverse	CTCTTCTTTCCAATTTTTTCCAAATAATC
cg06631999_Sequencing	CTCTAACTCAATCCCTAAATAC
cg23068797_Forward_Biotin	TTTTTGGGTTTAGGAGGAATGTT
cg23068797_Reverse	CCAACTAATACCACATCTAAAACTATTTACAATAC
cg23068797_Sequencing	AATACCACATCTAAAACTATTTAC
cg27309098_Forward_Biotin	GAGGAAATTGAGGTTTAGAGATATGAA
cg27309098_Reverse	CTAAAATCAAACTTAAAATACAACTCCTTAATA
cg27309098_Sequencing	CAAAAAAATTTTTACC
cg27197524_Forward_Biotin	GAAGAATTTGAATTTTAGGGAAGAAGTAT
cg27197524_Reverse	CCCAACAAACAAAAACAAAAATTCAATA
cg27197524_Sequencing	TACTAAACTCTAAAACCTAC
cg21548464_Forward	GTTTTTTAGTTGGGATTTATTTAGATTTGT
cg21548464_Reverse_Biotin	AACCCTTACCATCTTCTACCTAAACT
cg21548464_Sequencing	CATATTCAAAATTCTCATCAT
cg09998451_Forward_Biotin	GGGAATTTTGTATTTTAGTTGTGGATTTTT
cg09998451_Reverse	TAAACCTCAATTAACCCCTACTCAA
cg09998451_Sequencing	GTAAAATTTTGTTTGAT
